# A Remote Two-Point Magnetic Localization Method Based on SQUID Magnetometers and Magnetic Gradient Tensor Invariants

**DOI:** 10.3390/s24185917

**Published:** 2024-09-12

**Authors:** Yingzi Zhang, Gaigai Liu, Chen Wang, Longqing Qiu, Hongliang Wang, Wenyi Liu

**Affiliations:** 1 State Key Laboratory of Dynamic Measurement Technology, North University of China, Taiyuan 030051, China; zhangyingzi@nuc.edu.cn (Y.Z.); b200610@st.nuc.edu.cn (G.L.); b20220620@st.nuc.edu.cn (C.W.); wanghongliang@nuc.edu.cn (H.W.); 2 Shanghai Institute of Microsystem and Information Technology, Chinese Academy of Sciences, Shanghai 200050, China; lq.qiu@mail.sim.ac.cn

**Keywords:** magnetic localization, magnetic gradient tensor, superconducting quantum interference device, magnetic anomaly detection

## Abstract

In practical application, existing two-point magnetic gradient tensor (MGT) localization methods have a maximum detection distance of only 2.5 m, and the magnetic moment vectors of measured targets are all unknown. In order to realize remote, real-time localization, a new two-point magnetic localization method based on self-developed, ultra-sensitive superconducting quantum interference device (SQUID) magnetometers and MGT invariants is proposed. Both the magnetic moment vector and the relative position vector can be directly calculated based on the linear positioning model, and a quasi-Newton optimization algorithm is adopted to further improve the interference suppression capability. The simulation results show that the detection distance of the proposed method can reach 500 m when the superconducting MGT measurement system is used. Compared with Nara’s single-point tensor (NSPT) method and Xu’s two-point tensor (XTPT) method, the proposed method produces the smallest relative localization error (i.e., significantly less than 1% in the non-positioning blind area) without sacrificing real-time characteristics. The causes of and solutions to the positioning blind area are also analyzed. The equivalent experiments, which were conducted with a detection distance of 10 m, validate the effectiveness of the localization method, yielding a minimum relative localization error of 4.5229%.

## 1. Introduction

Since the magnetic characteristics of underwater targets are difficult to camouflage, magnetic anomaly detection (MAD) technology based on magnetic sensors has received a great deal of attention in the field of underwater target detection and localization. Compared with the traditional magnetic field scalar measurement and magnetic field vector measurement, magnetic gradient tensor (MGT) measurement can not only avoid the influence of the geomagnetic background field and the magnetization direction of the magnetic target, but it can also directly locate the magnetic dipole target through the MGT inversion algorithm [1,2,3]. As a result, MGT measurement and inversion has become a research hotspot in geophysical exploration, archaeology, and security applications [4,5,6,7].

The development of an ultra-sensitive MGT measurement system is essential for detecting the magnetic anomaly changes generated by long-distance underwater targets. MGT measurement systems can be constructed by fluxgates or superconducting quantum interference devices (SQUIDs) [8,9], with the detection distance is related to their sensitivity. As we all know, the sensitivity of SQUID magnetometers is 2 to 3 orders higher than that of fluxgate magnetometers [10]. Meanwhile, the length of the baseline of SQUID gradiometers is much smaller than that of fluxgate gradiometers under the same gradient sensitivity, which is conducive to the miniaturization of the design of measurement systems [11,12]. In addition, the bandwidth of SQUID magnetometers is much higher than that of fluxgate magnetometers, and it is more suitable for measurement occasions where the signal to be measured changes dramatically [13]. However, the current MGT measurement system, which is designed to realize the real-time localization of a single magnetic dipole target, is mainly constructed by fluxgate magnetometers, and the maximum detection distance is only 5 m. Therefore, an MGT measurement system based on SQUID magnetometers was designed in this paper to improve detection distance.

The relative position vector from the observation point to the magnetic dipole object can be calculated using the measured MGT and its invariants, a process called MGT inversion. According to the number of observation points, the current magnetic localization methods based on MGT inversion algorithms are divided into two categories: single-point tensor (SPT) methods and two-point tensor (TPT) methods. SPT methods can be categorized into three main groups: eigenvalue-based localization methods, Nara’s single-point tensor (NSPT) methods, and scalar triangulation and ranging (STAR) methods. Eigenvalue-based localization methods were developed rapidly after Wynn [14,15] and Frahm [16] proposed and promoted the innovative application of MGT for point-to-point positionings of magnetic dipoles [17,18]. However, the solution of this method has an inherent fourfold ambiguity; it requires adding a measuring point or additional information to eliminate ‘ghost’ solutions [19]. The NSPT method, which only needs to measure the MGT and the magnetic field vector at one observation point [20], has attracted a great deal of attention because of its simplicity and high real-time performance. In order to solve the localization dead-zone problem of this method, the Moore–Penrose generalized inverse matrix [21], truncated singular-value decomposition [22], and eigenvector constraint-based method [23] were studied to eliminate the MGT singularity at certain measuring points. However, the above-mentioned NSPT-based methods all involve the measurement of the magnetic field vector of a magnetic dipole target, which is difficult to separate from the geomagnetic field in the actual measurement process, and a small geomagnetic field measurement error leads to a considerable localization error. Certain single-point, high-order MGT localization methods based on NSPT have been researched [24,25], but they are more susceptible to instrument measurement errors. The STAR method, based on tensor invariants, which has also been widely studied, was proposed in order to eliminate the influence of the geomagnetic background field [26,27,28,29,30]. Additionally, it has a unique advantage in highly dynamic magnetic detection applications. However, the inherent aspherical error and greater number of instrument measurement errors introduced by the eight triaxial fluxgate magnetometers contained in the probe structure reduce the positioning accuracy of this method. As a result, the TPT method was gradually developed; it is not affected by the geomagnetic field and does not have aspherical errors. The TPT methods proposed earlier either required prior information [3] or adopted optimization algorithms, such as particle swarm optimization (PSO) [31]. These are complex in computation and relatively poor in real-time performance. Compared to them, Xu’s two-point tensor (XTPT) method [2] can provide an analytical solution of the relative position vector that is simple and provided in real time. However, an approximation error is introduced if the distance between the two observation points is not sufficiently small. Therefore, our research group proposed a new two-point tensor (NTPT) method [32] with higher localization accuracy, which is not only unaffected by the geomagnetic field, but also has no approximate errors. In this method, a new simulation model was established that takes into account the influence of different z-coordinates on localization. However, the NTPT method only considers cases where the relative position vectors and the magnetic moment vector are not coplanar and the noise suppression ability needs to be enhanced in the actual experiment process.

In this paper, a two-point magnetic gradient tensor (TMGT) localization method for remote, single-magnetic-dipole targets based on SQUID magnetometers and MGT invariants is proposed. In order to improve the detection distance, an MGT probe composed of eight self-developed SQUID magnetometers was designed. A linear localization model based on the spatial position relationship between a magnetic moment vector and relative position vectors—which can not only realize the high-precision localization of magnetic target, but also calculate its magnetic moment vector—is also proposed. At the same time, an objective function based on the relationship between the measured MGTs and the relative position vectors was constructed to optimize the inversion results, and the magnetic interference suppression ability was further improved. A long-distance spherical trajectory simulation model was established to analyze the positioning accuracy and positioning blind area of this method. Both simulations and equivalent experiments helped to demonstrate that the TMGT method has a better localization performance for remote magnetic targets.

## 2. Methods

### 2.1. Magnetic Gradient Tensor and Tensor Invariants

The magnetization of a magnetic target by the geomagnetic field causes a local distortion in the geomagnetic field, thus forming a magnetic anomaly. The aim behind the detection of magnetic anomalies is to locate the target by detecting these distortions. The magnetic target can be equivalent to a magnetic dipole when the detection distance is greater than 2.5 times the maximum size of the magnetic target. According to the Biot–Savart law, the magnetic induction intensity vector $B=(B_{x},B_{y},B_{z})$ generated by a magnetic dipole at a certain observation point can be expressed as follows:

(1) $$B=\frac{\mu }{4\pi }\frac{3\left(M\cdot r\right)r-Mr^{2}}{r^{5}}=\frac{\mu }{4\pi r^{5}}\left[\begin{array}{ccc} 3r_{x}^{2}-r^{2} & 3r_{x}r_{y} & 3r_{x}r_{z}\\ 3r_{x}r_{y} & 3r_{y}^{2}-r^{2} & 3r_{y}r_{z}\\ 3r_{x}r_{z} & 3r_{y}r_{z} & 3r_{z}^{2}-r^{2} \end{array}\right]\left[\begin{array}{c} M_{x}\\ M_{y}\\ M_{z} \end{array}\right],$$

where $r=(r_{x},r_{y},r_{z})$ is the relative position vector from the magnetic dipole to the observation point, *r* is the modulus of r, $M=(M_{x},M_{y},M_{z})$ is the magnetic moment vector of the magnetic dipole, and μ is the permeability of the medium at the observation point. Since the observation point is usually in the air, then $\mu \approx \mu_{0}=4\pi \times 10^{-7}N\cdot A^{-2}$, where $\mu_{0}$ is the vacuum permeability.

The magnetic gradient tensor G is the spatial variation rate of the three components of the magnetic induction intensity vector B in the orthogonal direction, and it has nine elements in total. Among them, the curl and divergence of the magnetic field vector at a certain point in the passive static magnetic field are zero [32]. Hence, the magnetic gradient tensor G has only five independent elements and can be denoted as follows:

(2) $$G=\left[\begin{array}{c} B_{x}\\ B_{y}\\ B_{z} \end{array}\right]\left[\begin{array}{ccc} \frac{\partial }{\partial x}\; & \frac{\partial }{\partial y}\; & \frac{\partial }{\partial z} \end{array}\right]=\left[\begin{array}{ccc} B_{xx} & B_{xy} & B_{xz}\\ B_{xy} & B_{yy} & B_{yz}\\ B_{xz} & B_{yz} & -B_{xx}-B_{yy} \end{array}\right].$$

The five independent elements of G are expressed as follows:

(3) $$\left[\begin{array}{c} B_{xx}\\ B_{xy}\\ B_{xz}\\ B_{yy}\\ B_{zz} \end{array}\right]=\frac{\mu }{4\pi r^{7}}\left[\begin{array}{ccc} \left(9r^{2}-15r_{x}^{2}\right)r_{x}\; & \left(3r^{2}-15r_{x}^{2}\right)r_{y}\; & \left(3r^{2}-15r_{x}^{2}\right)r_{z}\\ \left(3r^{2}-15r_{x}^{2}\right)r_{y}\; & \left(3r^{2}-15r_{y}^{2}\right)r_{x}\; & -15r_{x}r_{y}r_{z}\\ \left(3r^{2}-15r_{x}^{2}\right)r_{z}\; & -15r_{x}r_{y}r_{z}\; & \left(3r^{2}-15r_{z}^{2}\right)r_{x}\\ \left(3r^{2}-15r_{y}^{2}\right)r_{x}\; & \left(3r^{2}-15r_{y}^{2}\right)r_{y}\; & \left(3r^{2}-15r_{y}^{2}\right)r_{z}\\ -15r_{x}r_{y}r_{z}\; & \left(3r^{2}-15r_{y}^{2}\right)r_{z}\; & \left(3r^{2}-15r_{z}^{2}\right)r_{y} \end{array}\right]\left[\begin{array}{c} M_{x}\\ M_{y}\\ M_{z} \end{array}\right].$$

The eigenvalues of the characteristic equation of G can be derived as follows [18]:

(4) $$\left\{\begin{array}{c} \lambda_{1}=\frac{3\mu_{0}M}{8\pi r^{4}}\left(-\cos\theta +\sqrt{5{\left(\cos\theta \right)}^{2}+4}\right)\\ \lambda_{2}=\frac{3\mu_{0}M}{4\pi r^{4}}\cos\theta \\ \lambda_{1}=\frac{3\mu_{0}M}{8\pi r^{4}}\left(-\cos\theta -\sqrt{5{\left(\cos\theta \right)}^{2}+4}\right) \end{array}\right.,$$

where $M=∥M∥$ is the modulus of M, and θ is the angle between r and M. From Equation (3), cosθ can be deduced and denoted as follows:

(5) $$\cos\theta =\frac{M\cdot r}{∥M∥\cdot ∥r∥}=\frac{\lambda_{2}}{\sqrt{-\lambda_{2}^{2}-\lambda_{1}\lambda_{3}}},$$

and the relationships between the three eigenvalues above are

(6) $$\left\{\begin{array}{c} \lambda_{1}+\lambda_{2}+\lambda_{3}=0\\ \lambda_{1}\geq \lambda_{2}\geq \lambda_{3}\\ \left|\lambda_{1}\right|\geq \left|\lambda_{2}\right|\\ \left|\lambda_{3}\right|\geq \left|\lambda_{2}\right| \end{array}\right..$$

These eigenvalues of G, and any combination of them, which are called tensor invariants, are independent of the coordinate system choice and are kept unchanged when the attitude of the magnetic gradient tensor measurement system changes. Therefore, tensor invariants are resistant to motion noise.

As the magnetic anomalies caused by magnetic dipoles have positive and negative peaks, the normalized source strength (NSS), a tensor invariant, is often used to locate the magnetic target because it is isotropic around the magnetic dipole and is not affected by the direction of magnetization. The NSS can be expressed as [18]

(7) $$NSS=\sqrt{-\lambda_{2}^{2}-\lambda_{1}\lambda_{3}}=\frac{3\mu_{0}M}{4\pi r^{4}}.$$

The three eigenvectors corresponding to the three real eigenvalues are expressed as follows:

(8) $$u_{i}=\left[\begin{array}{c} \alpha_{i}\\ \beta_{i}\\ \gamma_{i} \end{array}\right]=\left[\begin{array}{c} B_{yz}B_{xy}+\left(\lambda_{i}-B_{yy}\right)B_{xz}\\ B_{xz}B_{xy}+\left(\lambda_{i}-B_{xx}\right)B_{yz}\\ \left(B_{xx}-\lambda_{i}\right)\left(B_{yy}-\lambda_{i}\right)-B_{xy}^{2} \end{array}\right],\;i=1,2,3,$$

which are orthogonal to each other. In the above equation, $n_{i}=u_{i}/\left|u_{i}\right|$ is the corresponding unit eigenvectors, and $n_{2}$ is the unit normal vector unit of the plane defined by M and r, corresponding to the eigenvalue $\lambda_{2}$ with the smallest absolute value.

As a result, only five independent elements of G need to be measured, and the magnetic target can be located with the inversion algorithm based on the magnetic gradient tensor invariant.

### 2.2. Superconducting MGT Measurement System

**Figure 1:**
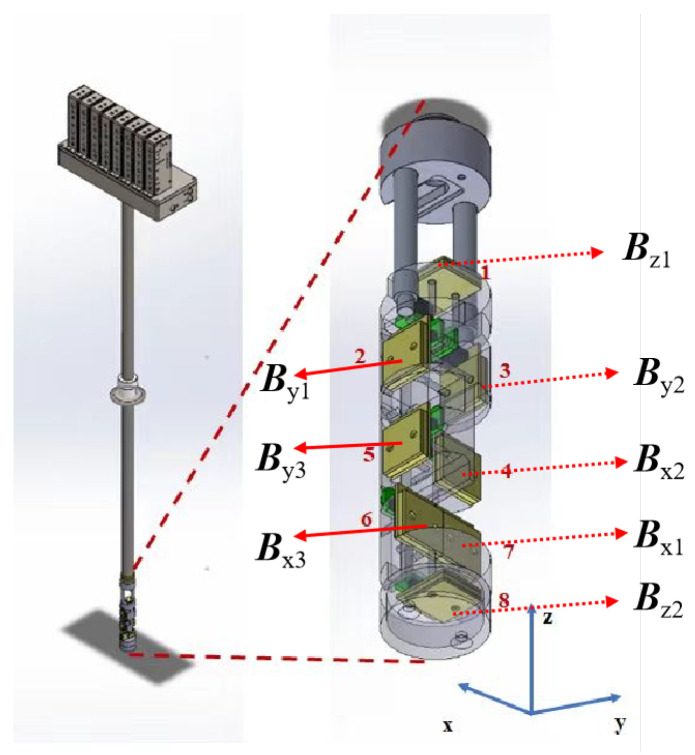
A structure diagram of the probe.

The superconducting MGT measurement system is mainly composed of an MGT probe, a non-magnetic liquid helium dewar, SQUID readout circuits, a data acquisition system, and a control software. The structure diagram of the probe is shown in Figure 1, and it is mainly composed of eight uniaxial SQUID magnetometers and one stainless steel probe rod. These SQUID magnetometers are divided into three groups, corresponding to the three axes of the Cartesian coordinate system, and the magnetometers are designed in the "face to face" form in order to construct an MGT measurement probe. When obtaining the MGT, the difference calculation is often used to replace the differential calculation in the MGT. As a result, the output of this system is expressed as follows:

(9) $$G=\left[\begin{array}{ccc} \frac{-\left(B_{y1}-B_{y2}\right)}{d_{23}}-\frac{\left(B_{z1}-B_{z2}\right)}{d_{18}} & \frac{B_{x1}-B_{x3}}{d_{67}} & \frac{B_{x1}-B_{x2}}{d_{47}}\\ \frac{B_{x1}-B_{x3}}{d_{67}} & \frac{B_{y1}-B_{y2}}{d_{23}} & \frac{B_{y1}-B_{y3}}{d_{25}}\\ \frac{B_{x1}-B_{x2}}{d_{47}} & \frac{B_{y1}-B_{y3}}{d_{25}} & \frac{B_{z1}-B_{z2}}{d_{18}} \end{array}\right],$$

where $B_{ij}$ represents the output of the SQUID magnetometers, *i* indicates the direction of the magnetometers (i=x,y,z), *j* indicates the number of magnetometers in a certain direction, and $d_{mn}$ indicates the distance between any two magnetometers in a probe.

### 2.3. Inversion Algorithm Based on MGT Invariants

**Figure 2:**
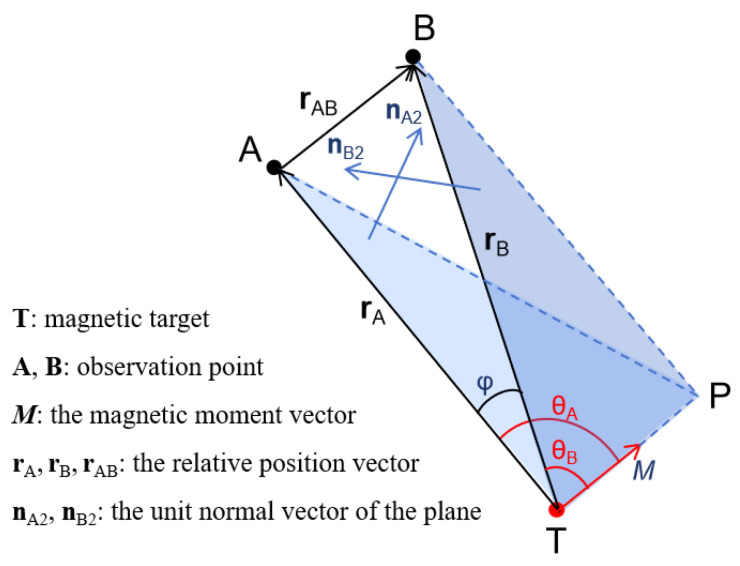
A schematic diagram of the non-coplanar inversion algorithm.

A schematic diagram of the inversion algorithm based on the two-point MGT proposed in this paper is shown in Figure 2, where *T* represents the magnetic target; *A* and *B* represent the two observation points; $r_{A}$ and $r_{B}$ are the relative position vectors of the magnetic target to observation point *A* and observation point *B*, respectively; φ is the angle between $r_{A}$ and $r_{B}$; M is the magnetic moment vector of the magnetic target; $\theta_{A}$ is the angle between $r_{A}$ and M; $\theta_{B}$ is the angle between $r_{B}$ and M; $n_{A2}$ is the unit normal vector of the plane ATP formed by M and $r_{A}$; and $n_{B2}$ is the unit normal vector of the plane BTP formed by M and $r_{B}$.

As can be seen from Figure 2, the relative position vector $r_{AB}$ from observation point *A* to observation point *B* can be expressed as follows:

(10) $$r_{AB}=r_{B}-r_{A}.$$

In the actual measurement process, since the positions of observation points *A* and *B* were known, the position of the magnetic target *T* could be obtained as long as $r_{A}$ or $r_{B}$ was solved. According to the law of cosine, the relationship between $r_{A}$ and $r_{B}$ can be denoted as follows:

(11) $$r_{AB}^{2}=r_{A}^{2}+r_{B}^{2}-2r_{A}r_{B}\cos\varphi ,$$

where $r_{A}$, $r_{B}$, and $r_{AB}$ are the moduli of $r_{A}$, $r_{B}$, and $r_{AB}$, respectively. According to Equation (6), the relation between $r_{A}$ and $r_{B}$ can also be deduced as follows:

(12) $$\frac{r_{A}^{4}}{r_{B}^{4}}=\frac{NSS_{B}}{NSS_{A}}.$$

As the magnetic gradient tensor G at the observation point can be obtained via the superconducting MGT measurement system, the NSS of the observation point can be calculated through the eigenvalues of *G*. Therefore, as long as cosφ is solved, $r_{A}$ can be obtained. The procedure for solving cosφ is as follows:

(a) When $n_{A2}\times n_{B2}\neq 0$, which means M is not coplanar with $r_{A}$ and $r_{B}$, the three vectors can form a triangular pyramid, as shown in Figure 2. Since the distance between observation points *A* and *B* is particularly small compared to the distance between the observation point and the magnetic target, φ and the dihedral angle α (the angle between the plane ATP and BTP) must be less than 90 degrees. According to the relation between the angle of the lines and the angle of the planes in the triangular pyramid, cosφ can be deduced as follows:

(13) $$\cos\varphi =\cos\theta_{A}\cos\theta_{B}+\sin\theta_{A}\sin\theta_{B}\cos\alpha ,$$

where $\cos\theta_{A}$ and $\cos\theta_{B}$ can be calculated from Equation (4). The value of $\sin\theta_{A}\sin\theta_{B}$ can be uniquely determined as both $\theta_{A}$ and $\theta_{B}$ are less than 180 degrees. Based on the relation between the dihedral angle α and the unit normal vector of the two planes, as well as considering the fact that α is an acute angle, cosα can be expressed as follows:

(14) $$\cos\alpha =\left|n_{A2}\cdot n_{B2}\right|.$$

**Figure 3:**
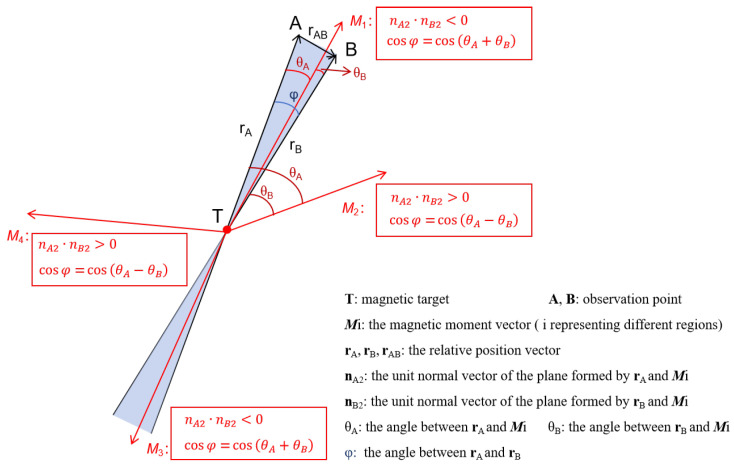
A schematic diagram of the coplanar localization algorithm.

(b) When $n_{A2}\times n_{B2}=0$, M is coplanar with $r_{A}$ and $r_{B}$, as shown in Figure 3, then $M_{i}$ is the magnetic moment vector of the magnetic dipole, where *i* represents different regions. If $n_{A2}\cdot n_{B2}<0$, which means that M is located at the acute angle area formed by the relative position vectors $r_{A}$ and $r_{B}$, then cosφ can be solved by

(15) $$\cos\varphi =\cos\left(\theta_{A}+\theta_{B}\right)=\cos\theta_{A}\cos\theta_{B}-\sin\theta_{A}\sin\theta_{B}.$$

If $n_{A2}\cdot n_{B2}>0$, which means the relative position vectors $r_{A}$ and $r_{B}$ are on the same side of M, then cosφ can be solved by

(16) $$\cos\varphi =\cos\left(\theta_{A}-\theta_{B}\right)=\cos\theta_{A}\cos\theta_{B}+\sin\theta_{A}\sin\theta_{B},$$

where $r_{A}$ can be calculated through Equations (11)–(13). Then, *M*, the modulus of the magnetic moment, can be solved by substituting $r_{A}$ into Equation (7).

The cosine of the angle between $r_{A}$ and $r_{B}$ can also be expressed as follows:

(17) $$\cos\varphi =\frac{r_{A}\cdot r_{B}}{r_{A}r_{B}}=\frac{r_{A}\cdot \left(r_{A}+r_{AB}\right)}{r_{A}r_{B}}=\frac{r_{A}^{2}+r_{A}n_{A}\cdot r_{AB}}{r_{A}r_{B}},$$

where $n_{A}$ is the unit direction vectors of $r_{A}$. According to the properties of the plane unit normal vector, the following equations can be obtained:

(18) $$n_{A}\cdot n_{A2}=0,$$

(19) $$n_{B}\cdot n_{B2}=\left(n_{A}+n_{AB}\right)\cdot n_{B2}=0,$$

(20) $$M\cdot n_{A2}=Mn_{M}\cdot n_{A2}=0,$$

(21) $$M\cdot n_{B2}=Mn_{M}\cdot n_{B2}=0,$$

where $n_{B}$, $n_{AB}$, and $n_{M}$ are the unit direction vectors of $r_{B}$, $r_{AB}$, and M. By combining Equations (5) and (17), $n_{A}$, $n_{B}$, and $n_{M}$ can be solved. As a result, $r_{A}$, $r_{B}$, and M can be obtained via the following:

(22) $$r_{A}=r_{A}n_{A},$$

(23) $$r_{B}=r_{B}n_{B},$$

(24) $$M=Mn_{M}.$$

However, in the actual detection process, the $r_{A}$ calculated by this algorithm is accurate only when the signal-to-noise ratio is particularly high. Therefore, due to the presence of interference, the solution of $r_{A}$ is transformed into an optimization problem. In order to improve the anti-interference ability of this localization method, the objective function can be constructed as follows [2]:

(25) $$f=\min{∥\left(G_{A}-G_{B}\right)n_{A}-\left(3G_{A}+G_{B}\right)r_{\mathrm{AB}}∥}_{2},$$

where $G_{A}$ and $G_{B}$ are the magnetic gradient tensor of observation points A and B, respectively. The relative position vector $r_{A}$ obtained from Equation (22) is taken as the initial solution, and a more accurate $r_{A}$ can be solved via the quasi-Newton optimization algorithm. Naturally, the magnetic moment of the magnetic dipole M can be obtained via the final $r_{A}$.

As a result, in the whole inversion process, only the positions of two observation points and the corresponding magnetic gradient tensors are needed in the TMGT algorithm, which is not affected by the geomagnetic field and has strong anti-interference ability.

## 3. Simulations

**Figure 4:**
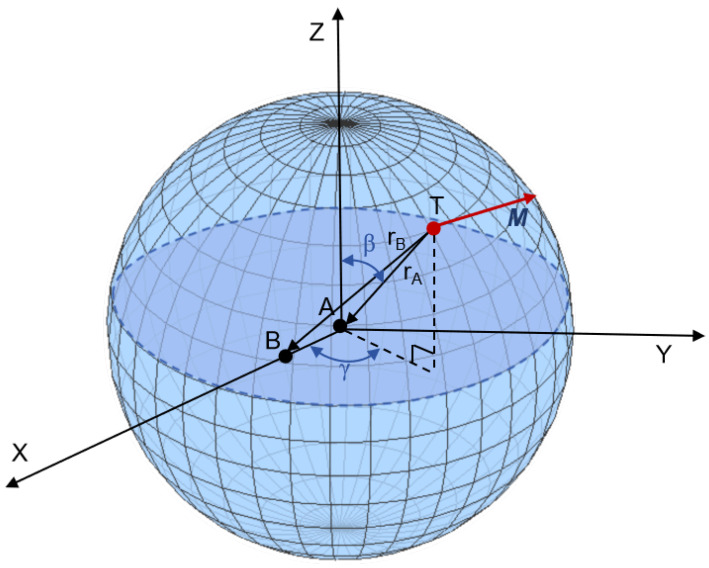
Spherical trajectory model.

As shown in Figure 4, a spherical trajectory model was established to analyze the performance of the algorithms under various orientations of the magnetic target in order to verify the feasibility of the TMGT localization algorithm. In this model, observation point A is at the origin of the coordinate system and observation point B is 10 m away from observation point A on the x-axis. The magnetic target T moves along the surface of the spherical trajectory model, where the polar angle β varies from 0 to 180 degrees and the azimuthal angle γ varies from 0 to 360 degrees (both with an interval of 1 degree). The radius $r_{A}$ of the model is 500 m, and the magnetic moment M of the magnetic target T is (50, 50, 70.7) × $10^{6}$ A·m^2^. In order to quantitatively evaluate the localization performance of the inversion algorithm, the relative localization error ε is used and defined as

(26) $$\varepsilon =\sqrt{{\left(-x_{t}+x_{t0}\right)}^{2}+{\left(-y_{t}+y_{t0}\right)}^{2}+{\left(-z_{t}+z_{t0}\right)}^{2}}/r_{A}\times 100\%,$$

where ($x_{t0}$, $y_{t0}$, $z_{t0}$) is the real coordinate of the magnetic target, and ($x_{t}$, $y_{t}$, $z_{t}$) is the estimated coordinate of the inversion algorithm. During the remote magnetic target localization process, if ε exceeds 10%, then the localization is considered a failure.

### 3.1. Without the Influence of Noise

**Figure 5:**
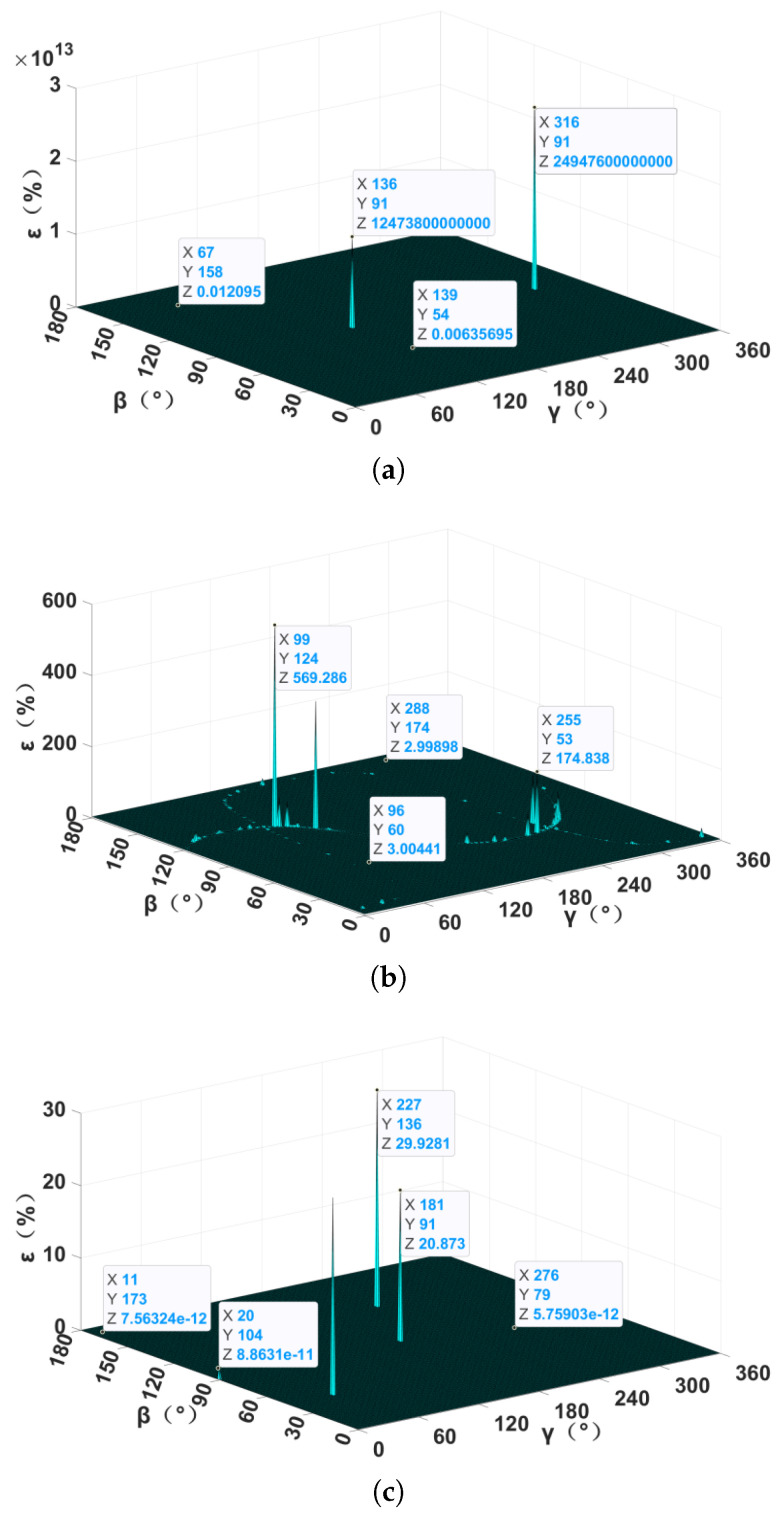
Relative localization error without noise: (**a**) NSPT method; (**b**) XTPT method; (**c**) TMGT method.

In order to compare the localization performance of the NSPT, XTPT, and TMGT methods, a set of simulations without noise were carried out, and the simulation results are shown in Figure 5. It can be seen that the relative localization errors were particularly large when the magnetic target was at certain points, which can be called positioning blind spots. Without the influence of noise, the location and number of the positioning blind spots in each method were kept unchanged after repeated simulations. Among these three methods, XTPT had the largest number of positioning blind spots. TMGT had three positioning blind spots, and NSPT had two positioning blind spots. In the non-positioning blind area, the relative localization error of the TMGT method was much smaller than those of the NSPT and XTPT methods, which were at about a magnitude of $10^{-12}$ or below. Meanwhile, the relative localization error of the NSPT method was three orders of magnitude smaller than that of the XTPT method. In summary, under ideal conditions, the TMGT method proposed in this paper had the best localization performance, followed by the NSPT and XTPT methods.

### 3.2. With the Influence of Noise

Magnetic field measurement noise, which mainly includes geomagnetic background measurement noise and instrument measurement noise, are unavoidable in practical localization applications. Since the slightest geomagnetic activity could cause magnetic field fluctuations of ±20 nT, a Gaussian distribution geomagnetic noise with a mean of 0 and a standard deviation of 20 nT was added to the three components of the calculated magnetic induction vector B. At the same time, instrument measurement noise with an average of 0 and a standard deviation of $10^{-13}$ T was added to each element of the calculated magnetic gradient tensor G since the sensitivity of the superconducting MGT measurement system, which is determined by the bottom line of magnetic noise, was generally in the order of $10^{-13}$ T. Then, a set of simulations involving magnetic field measurement noise was carried out.

**Figure 6:**
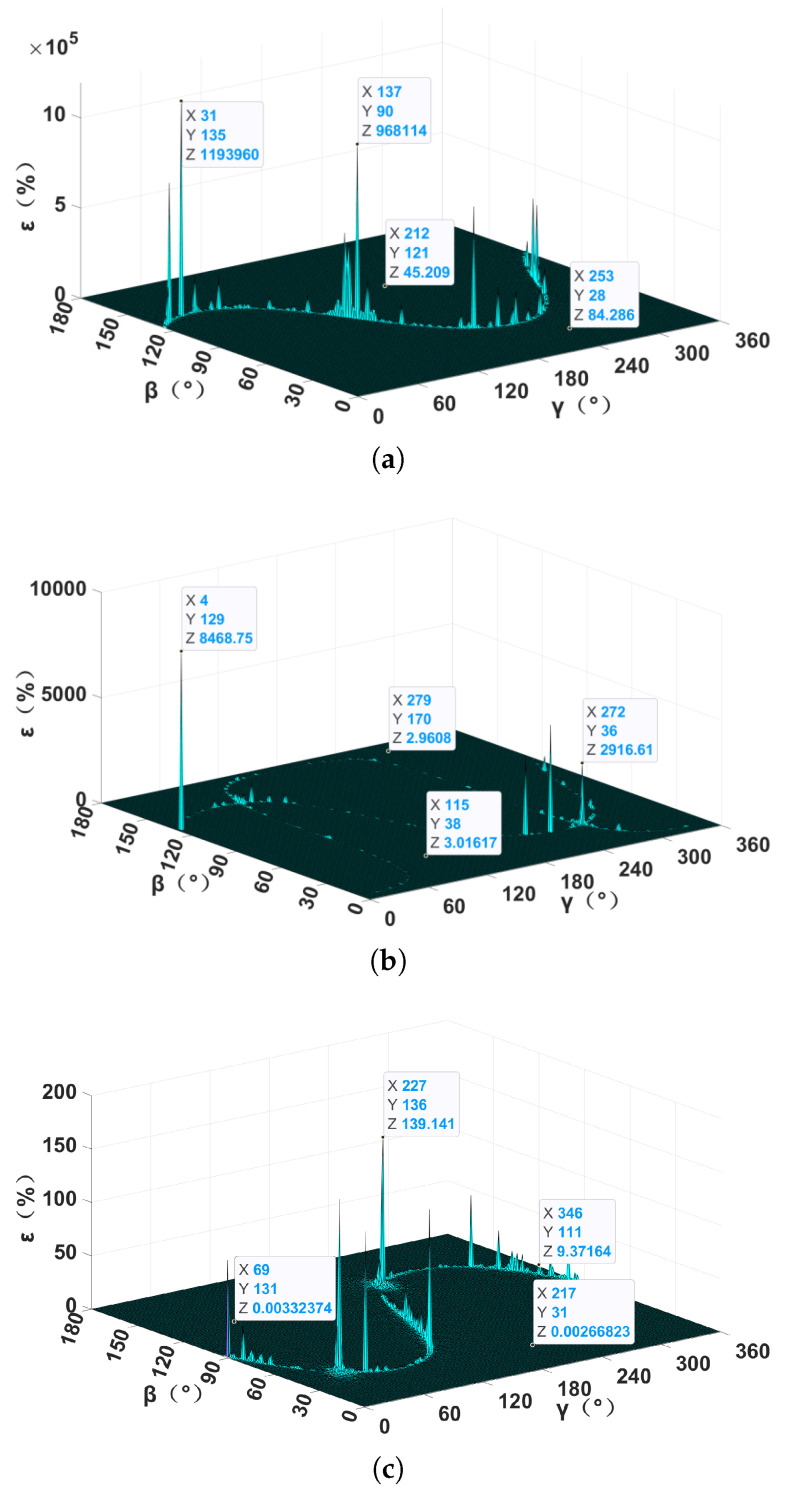
The relative localization error with noise: (**a**) NSPT method; (**b**) XTPT method; and (**c**) TMGT method.

As illustrated in Figure 6, under the same simulation conditions, each method had its own positioning blind area, and the location of the area was kept unchanged in repeated simulations. In these three methods, the positioning blind areas of the TMGT method and NSPT method were distributed like a sine wave curve, while that of the XTPT method was distributed along two curves and had the largest number of positioning blind spots. In the non-positioning blind area, the NSPT method needs to measure the magnetic induction intensity vector B at the observation point caused by the magnetic target, which is inversely proportional to the third power of the detection distance. When the detection distance was 500 m, the magnetic induction intensity vector signal generated by the magnetic target was submerged in the background noise of the geomagnetic field, and the relative localization errors were greater than 10%, resulting in a failure of positioning. As the TMGT and XTPT methods only need to measure the MGT at the two observation points, they were mainly affected by the instrument measurement noise. With the same instrument measurement noise, the relative localization error of the XTPT method was about 3% in the non-positioning blind area with or without of noise, and the relative localization error of the TMGT method was two to three orders smaller than that of the XTPT method. Compared with the other two methods, the TMGT method has better noise resistance and localization accuracy.

**Figure 7:**
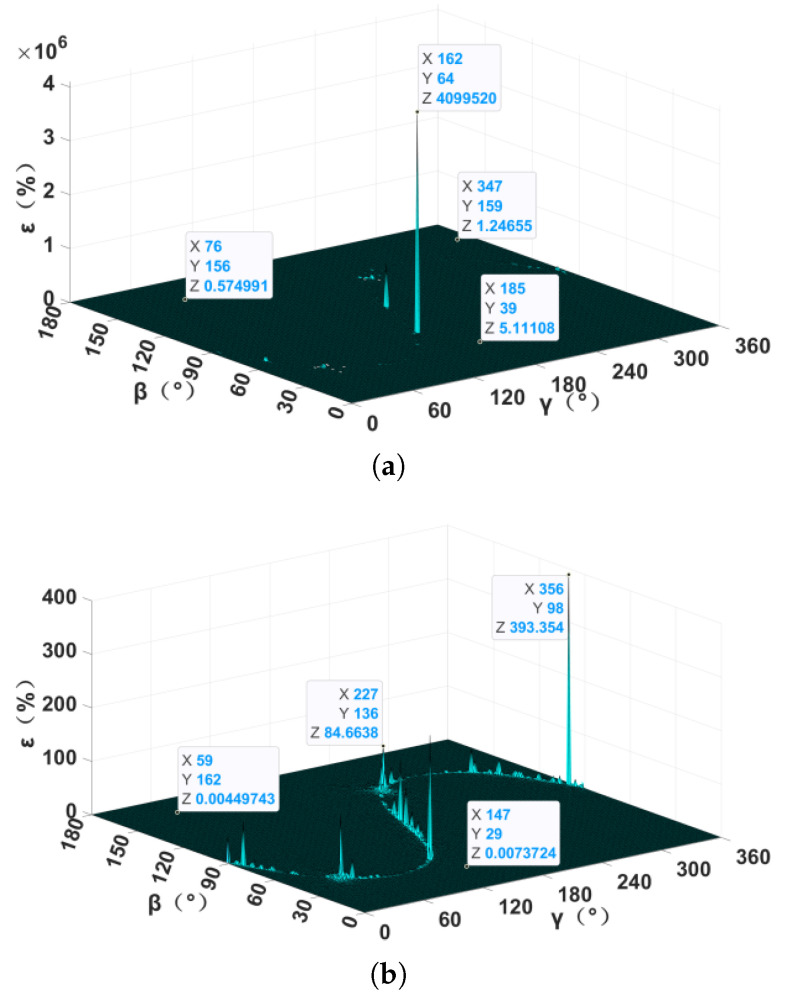
The relative localization error with different instrument noise: (**a**) standard deviation of $10^{-12}$ T; (**b**) standard deviation of $10^{-14}$ T.

The influence of different instrument noise on the localization error of the TMGT method was clear when the detection distance and magnetic moment were the same, as shown in Figure 7. Evidently, the greater the instrument noise, the greater the localization error and vice versa. It can be inferred that under the same magnetic moment and localization error, the smaller the instrument noise, the farther the detection distance. Therefore, the probe structure and readout circuits of the measurement system must be optimized to reduce the instrument noise of the whole system in order to improve the positioning accuracy or detection distance of the measuring system.

### 3.3. Positioning Blind-Area Analysis

**Figure 8:**
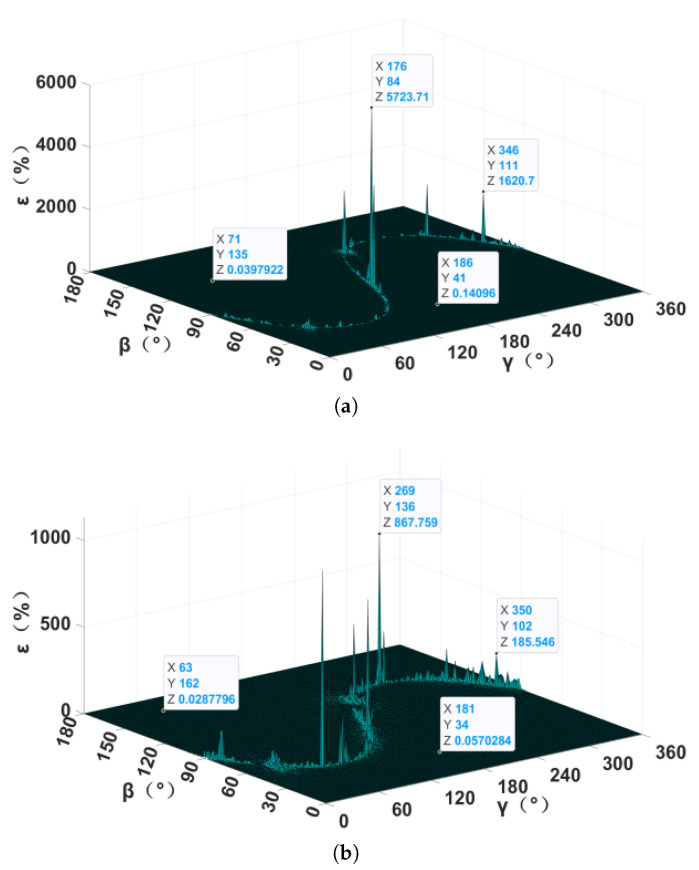
Relative localization error with different magnetic moment vectors: (**a**) M = (50, 70.7, 50) × $10^{6}$ A·m^2^; (**b**) M = (70.7, 50, 50) × $10^{6}$ A·m^2^.

When the magnetic target is located at some points, the MGT matrix at the observation points is singular, and the area where these points converge is called the positioning blind area. Although the TMGT method does not calculate the inverse of the MGT, it may influence the solving of the spatial positioning model. In the previous simulation, it was found that different localization methods have their own fixed positioning blind area under the fixed magnetic moment vectors of a magnetic target. In order to further verify this conjecture, a simulation that only changed the magnetic moment vector was carried out, the results of which are shown in Figure 8. Different magnetic moment vectors were found to only change the relative localization error in the positioning blind area. The location of the positioning blind area was kept unchanged, and the localization performance in the non-positioning blind area was not affected. In order to eliminate the effect of the irreversible MGT matrix, the Moore–Penrose generalized inverse matrix [21], truncated singular value decomposition [22], or eigenvector constraint-based method [23] can be adopted. In practical detection applications, multiple localization methods or measurement systems can also be combined to eliminate the positioning blind area.

### 3.4. With and without the Optimization Algorithm

**Figure 9:**
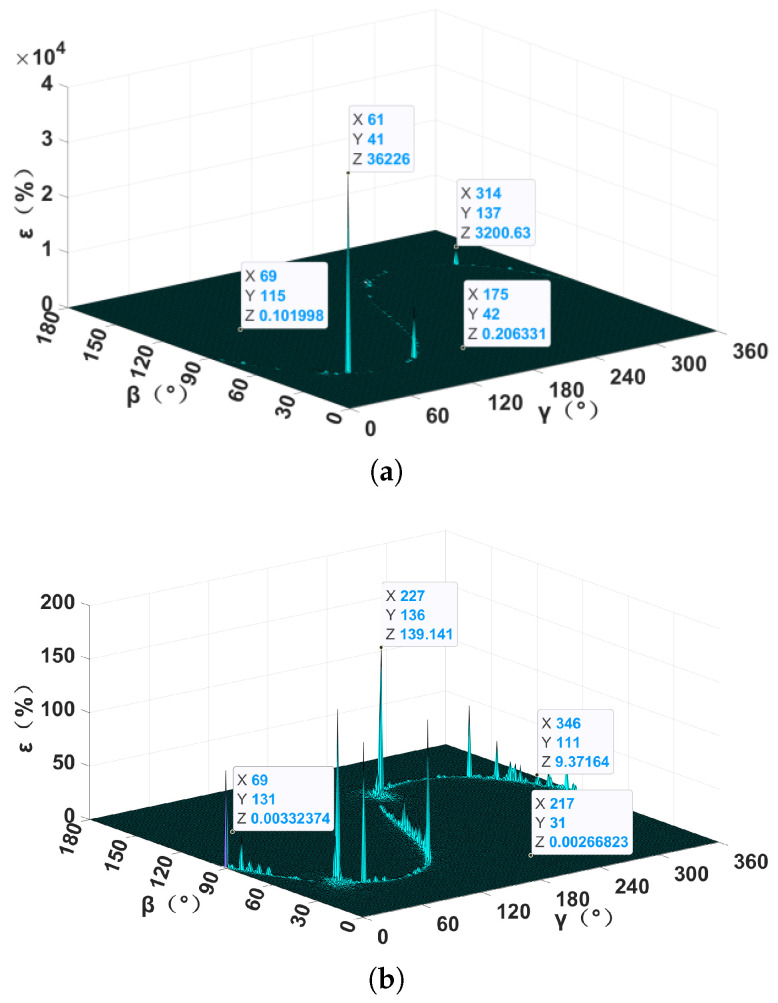
Relative localization error of the (**a**) NTPT method and (**b**) TMGT method.

The quasi-Newton optimization algorithm was added to the TMGT method in order to further improve the noise resistance, and the NTPT method was selected as a comparison in order to analyze the effect of the optimization algorithm on the localization performance. As shown in Figure 9, the relative localization error of the TMGT method was less than 10% at most points in the positioning blind area. Conversely, the relative localization errors of the NTPT method in the blind area were almost all greater than 10%. At the same time, the relative localization error of the TMGT method was about two orders of magnitude smaller than that of the NTPT method in the non-positioning blind area.

Since the introduction of a non-linear optimization algorithm will generally increase the localization time, the average localization time of the TMGT method and NTPT method were also compared and analyzed, and the maximum number of iterations of the TMGT method was set to 1000. The simulation code was performed on a laptop equipped with an Intel ^®^ Core ™ Ultra 7 155H processor running at a nominal clock speed of 1.4 GHz. In each simulation cycle, the magnetic targets that moved along 65,341 points on the spherical trajectory model were localized. The total time required by the TMGT method was 1987.299689 s, and the average time for locating each point was about 0.030414 s. The total time of the NTPT method was 1999.893442 s, and the average time for locating each point was about 0.030607 s. It can be seen that the TMGT method proposed in this paper does not deteriorate in real time due to the introduction of an optimization algorithm, and it is also demonstrated that it can meet real-time localization requirements.

## 4. Experiments and Results Analysis

### 4.1. Equivalent Experimental Setup

**Figure 10:**
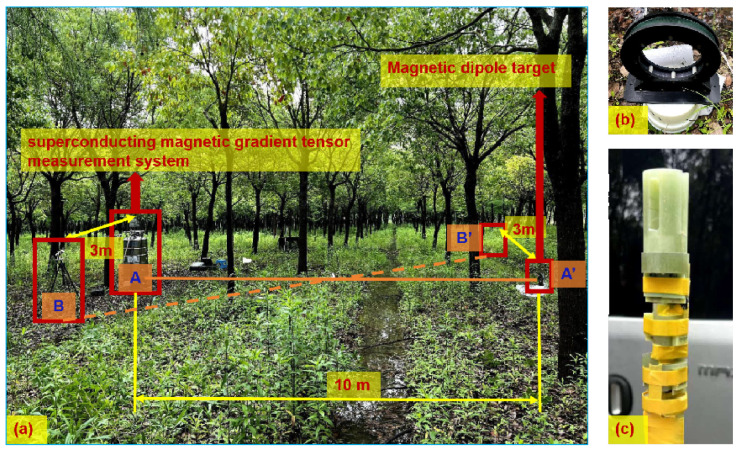
The equivalence experiment: (**a**) the experimental site; (**b**) the magnetic dipole target; and (**c**) the superconducting magnetic gradient tensor probe.

Since it is difficult to find a site that can meet the requirement of a detection distance of 500 m with few magnetic interference factors, an equivalent experiment was adopted to verify the effectiveness of the TMGT method. In Section 3, the magnetic induction intensity generated by the magnetic dipole target at observation point A was on the order of $10^{-10}$ T to $10^{-13}$ T. Therefore, as seen in Figure 10b, a one-dimensional coil was designed and made as the magnetic dipole target in the equivalence experiment, and it was able to produce a magnetic induction intensity of about $10^{-10}$ T at a distance of 10 meters. The design parameters of the equivalent magnetic dipole object are shown in Table 1.

The SQUID magnetometers, the SQUID readout circuits, and the control software of the superconducting MGT system used in the experiment were all independently developed by the Shanghai Institute of Microsystem and Information Technology. The sensitivity of the SQUID magnetometers was 5 fT/Hz^2^. The pendulum rate of the superconducting magnetic gradient tensor measurement system was 2 mT/s, and the bandwidth affected by the metal dewar was about 500 Hz. The experimental data measured by the superconducting MGT probe were stored in the data acquisition system (NI 9235) through the SQUID readout circuits. The probe is shown in Figure 10c, and it is described in detail in Section 2.2. The baseline distance between the SQUID sensors was determined, and the output of the probe was as follows:

(27) $$G=100\left[\begin{array}{ccc} -\frac{1}{2}\left(B_{1y}-B_{2y}\right)-\frac{1}{11}\left(B_{1z}-B_{2z}\right) & \frac{10}{17}\left(B_{1x}-B_{3x}\right) & \frac{5}{14}\left(B_{1x}-B_{2x}\right)\\ \frac{10}{17}\left(B_{1x}-B_{3x}\right) & \frac{1}{2}\left(B_{1y}-B_{2y}\right) & \frac{5}{16}\left(B_{1y}-B_{3y}\right)\\ \frac{5}{14}\left(B_{1x}-B_{2x}\right) & \frac{5}{16}\left(B_{1y}-B_{3y}\right) & \frac{1}{11}\left(B_{1z}-B_{2z}\right) \end{array}\right].$$

As shown in Figure 10, the site of this experiment was Hengsha Island in Shanghai, where the background magnetic field is relatively clean. Before the experiment, in order to further reduce the magnetic interference caused by vibration and other factors, we buried the superconducting MGT measurement system underground. Observation point A was set as the origin of the Cartesian coordinate system, and the positive direction of the x-axis was set as south on the compass. The coordinates of the magnetic target position $A^{\prime }$ were about (−10, 0, 1) m, the coordinates of observation point B were about (0, −3, 0) m, and the coordinates of the magnetic target position $B^{\prime }$ were about (−10, 3, 0) m. The TMGT method needs to measure the magnetic gradient tensor at two observation points, but there was only one superconducting magnetic gradient tensor measurement system. According to the relativity of measurement, the MGT at observation point B generated by the magnetic dipole target T at position $A^{\prime }$ is the same as the MGT at observation point A that is generated by the magnetic dipole target T at position $B^{\prime }$. Therefore, in order to facilitate the experiment—after measuring the magnetic gradient tensor $G_{A}$ at observation point A, which is generated by the magnetic dipole target T at the position $A^{\prime }$—we moved the magnetic dipole target T to position B’, and we then measured the magnetic gradient tensor $G_{B}$ at observation point A. Finally, the TMGT method and XTPT method were used to locate the magnetic target at the position $A^{\prime }$ based on the measured $G_{A}$ and $G_{B}$.

### 4.2. Analysis of Experimental Results

Compared with the other localization methods mentioned above, the TMGT method not only has higher localization accuracy but can also calculate the magnetic moment vector of the measured target. The higher the localization accuracy, the closer the magnetic moment vector is to the true value. Eight different currents were successively added to the one-dimensional coil to simulate the magnetic dipole target with different magnetic moments, and eight sets of measurement data were obtained. Due to the excessive, uncontrolled magnetic interference in the field experiment, the localization errors of the other two-point methods were all much greater than 10% in the eight experiments; as such, only the experimental results of the TMGT method are listed in Table 2. Although the superconducting MGT measurement system is highly susceptible to magnetic interference due to its ultra-high sensitivity, the TMGT method can still achieve a relative good localization effect when the current is greater than 1.242 A in the field experiment. The minimum relative localization error was 4.5229%, and the corresponding magnetic moment amplitude was 3.3940 × 10^10^ A·m^2^. The magnetic gradient tensor generated by the magnetic dipole target at the observation point was overwhelmed by the magnetic noise when the current was too low. Hence, the magnetic noise mechanism and compensation algorithm of the superconducting MGT measurement system will be studied in order to further reduce the influence of magnetic noise and improve the detection distance. At the same time, the localization method proposed in this paper was mainly designed for a single magnetic dipole target; thus, it is not applicable when there are multiple magnetic dipole targets or if the measurement distance is too small. Therefore, the positioning method will be further optimized for different magnetic source models [33,34,35,36] in the future.

## 5. Conclusions

At present, the MGT inversion algorithms are mainly applied to the MGT detection system based on fluxgate magnetometers. In this case, the detection distance is limited and the magnetic moment vector of the magnetic target cannot be calculated. In order to improve the detection range and obtain more information about the magnetic target, a remote magnetic localization method based on ultra-sensitive SQUID magnetometers and MGTs at two observation points was proposed. A spherical trajectory simulation model with a detection distance of 500 m was established in order to analyze the localization performance of the TMGT method for magnetic targets in any azimuth. The simulation results show the following: (a) the relative positioning error of the proposed TMGT method is much smaller than that of the NSPT method and the XTPT method regardless of noise; (b) the smaller the instrument measurement noise, the higher the localization accuracy and the farther the detection distance; (c) the positioning blind area of the TMGT method can be eliminated; and (d) the TMGT method, when using a quasi-Newton optimization algorithm, has better interference suppression ability and can meet the requirements of real-time detection. The equivalent test results show that the TMGT method not only has better localization performance in practical applications, but it can also calculate the magnetic moment vector of a magnetic dipole target. When the detection distance is 10 m, the minimum relative localization error of the TMGT method is 4.5229%. However, when the magnetic moment of the magnetic target is too small, the localization method cannot work. In the future, we will optimize the probe structure of the superconducting MGT system to reduce the instrument measurement noise so as to further improve the detection distance for the magnetic target.

## Figures and Tables

**Table 1:** The design parameters of the equivalent magnetic dipole object.

Name	Coil Specification	Coil Turns	Effective Diameter	Total Coil Inductance	Total Coil Resistance (25 °C)
One-dimensional coil	1.5 mm	150	200 mm	6.823 mH	0.93 Ω

**Table 2:** The experimental results of the TMGT method.

Sets	Current (A)	Relative Localization Error (%)	Magnetic Moment (A·m^2^)
1	0.828	338.5185	9.2624 × 10^12^
2	0.966	104.9234	3.8083 × 10^11^
3	1.104	42.2661	1.1361 × 10^11^
4	1.242	10.1966	2.4411 × 10^10^
5	1.380	4.5229	3.3940 × 10^10^
6	2.760	5.0574	2.8727 × 10^10^
7	4.140	6.0444	3.2539 × 10^10^
8	5.520	10.3482	3.0586 × 10^10^

## Data Availability

Dataset available on request from the authors (The raw data supporting the conclusions of this article will be made available by the authors on request.).
